# Large scale analysis of signal reachability

**DOI:** 10.1093/bioinformatics/btu262

**Published:** 2014-06-11

**Authors:** Andrei Todor, Haitham Gabr, Alin Dobra, Tamer Kahveci

**Affiliations:** CISE Department, University of Florida, Gainesville, FL 32611, USA

## Abstract

**Motivation:** Major disorders, such as leukemia, have been shown to alter the transcription of genes. Understanding how gene regulation is affected by such aberrations is of utmost importance. One promising strategy toward this objective is to compute whether signals can reach to the transcription factors through the transcription regulatory network (TRN). Due to the uncertainty of the regulatory interactions, this is a #P-complete problem and thus solving it for very large TRNs remains to be a challenge.

**Results:** We develop a novel and scalable method to compute the probability that a signal originating at any given set of source genes can arrive at any given set of target genes (i.e., transcription factors) when the topology of the underlying signaling network is uncertain. Our method tackles this problem for large networks while providing a provably accurate result. Our method follows a divide-and-conquer strategy. We break down the given network into a sequence of non-overlapping subnetworks such that reachability can be computed autonomously and sequentially on each subnetwork. We represent each interaction using a small polynomial. The product of these polynomials express different scenarios when a signal can or cannot reach to target genes from the source genes. We introduce polynomial collapsing operators for each subnetwork. These operators reduce the size of the resulting polynomial and thus the computational complexity dramatically. We show that our method scales to entire human regulatory networks in only seconds, while the existing methods fail beyond a few tens of genes and interactions. We demonstrate that our method can successfully characterize key reachability characteristics of the entire transcriptions regulatory networks of patients affected by eight different subtypes of leukemia, as well as those from healthy control samples.

**Availability:** All the datasets and code used in this article are available at bioinformatics.cise.ufl.edu/PReach/scalable.htm.

**Contact:** atodor@cise.ufl.edu

**Supplementary information:** Supplementary data are available at *Bioinformatics* online.

## 1 INTRODUCTION

Major disorders, such as cancer, have been shown to alter the transcription of a large number of genes and thus affect the mechanism that governs cells functions (Krivtsov, 2009; Valk *et al.*, 2004). Many complex disorders, such as acute lymphoblastic leukemias, however, yield a varying spectrum of expression profiles and, as a result, cannot be robustly characterized by merely studying the gene expressions (Armstrong, 2002).

An important part of cell biology research is the study of the causal relationship between extracellular conditions and the cell response. Such causality is governed by a chain of biochemical reactions through which extracellular signals are transmitted from membrane receptors to transcription factors (i.e., reporters) via protein–protein interactions (Bu and Callaway, 2011). While the pattern of this mechanism is similar for all organisms, important variations in its quantitative aspects such as gene expressions result from external perturbations, differentiation stage of the cell, timing of DNA replication and various epigenetic mutations (Los *et al.*, 2009; Mattick *et al.*, 2009). Therefore, detecting these quantitative variations is an important source of information for assessing the fitness of the organism and ultimately for diagnosis and prognosis.

Extensive evidence suggests that there is a degree of uncertainty in our knowledge of interactions within cells (Bader *et al.*, 2004; Ceol *et al.*, 2010; Deng *et al.*, 2003; Ourfali *et al.*, 2007; Sharan *et al.*, 2002; Suthram *et al.*, 2006; Szklarczyk *et al.*, 2011; von Mering *et al.*, 2002). The source of this uncertainty is 2-fold. First, the biological processes that are modeled as protein interactions in biological networks are stochastic events (Bader *et al.*, 2004). Second, the evidence in support of an interaction is not entirely decisive for the actual presence of the interaction (Bader *et al.*, 2004; Ourfali *et al.*, 2007; Sharan *et al.*, 2002; Shlomi *et al.*, 2006) due to many reasons, such as epigenetic variations across different cells (Gerstein *et al.*, 2012). Several schemes have already been proposed to assess the reliability of protein interactions in the form of confidence values (Bader *et al.*, 2004; Deng *et al.*, 2003; Suthram *et al.*, 2006). Such interaction confidence values are now available in large biological network databases, such as MINT (Ceol *et al.*, 2010) and STRING (Szklarczyk *et al.*, 2011).

Recent studies often model the uncertainty of the interactions in biological networks using probabilistic networks (Gabr *et al.*, 2013; Todor *et al.*, 2012; Todor *et al.*, 2013). We adopt the same model in this article, namely, each node of the network denotes a gene and the directed edge from a node *v_i_* to node *v_j_* denotes that the gene corresponding to *v_i_* can regulate the gene denoted by *v_j_* through an interaction. Each edge in this network is a probabilistic event. That is, it is considered possible, but not certain, reflecting the insecure knowledge of the gene regulation process. A common way to model the uncertainty of each edge is to associate it with a probability value, which is computed for each interaction from several factors: gene expressions, available evidence for it and network topology around it (Sharan *et al.*, 2002).

**Fig. 1.**
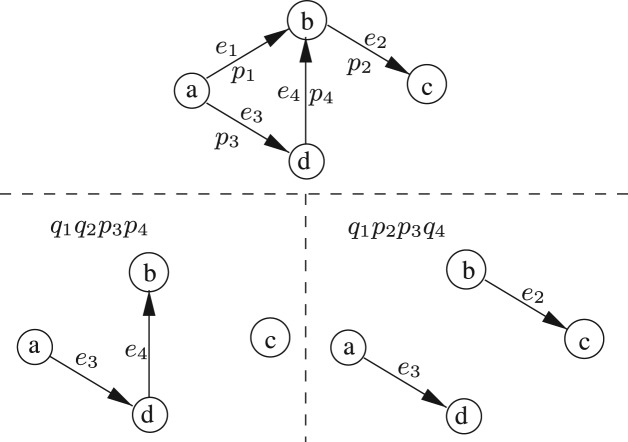
A probabilistic network (top) and two of the deterministic networks corresponding to it (bottom). Each of the deterministic networks is obtained from the probabilistic network with some probability determined by the probabilities of the edges that are included or not in the deterministic network. *p_i_* denotes the probability of edge *e_i_* being present. *q_i_* = 1– *p_i_* is the probability of the edge being absent. The expression above each deterministic network is the probability of observing it

The ability to compute confidence values for interactions provides opportunities to model and study biological networks accurately. It, however, comes at a high price as the uncertainty of the topology of interactions makes studying biological networks a computationally challenging task. The challenge is that a probabilistic network represents a large number of alternative deterministic network topologies. More precisely, a network with *n* probabilistic edges yields 2*^n^* possible network configurations, as each one of the *n* edges may be present or absent. For instance, in Figure 1, the probabilistic network shown on top corresponds to 16 deterministic networks since it contains 4 probabilistic edges.

In this article, we address the problem of characterizing the signaling reachability in transcription regulatory networks (TRNs). Unlike most of the existing literature, we eliminate the limitations of the classical assumption that all interactions are deterministic and adopt the more descriptive probabilistic network. More specifically, given a set of source genes $S=\{s_{1},s_{2},\ldots ,s_{a}\}$ and a set of target genes $T=\{t_{1},t_{2},\ldots ,t_{b}\}$, we compute the *reachability profile* of that network as a doubly indexed vector *R* where, for all *i*, *j* such that 1≤i≤a,1≤j≤b, the entry *R*[*i*,*j*] is the probability that a signal originating at *s_i_* can reach *t_i_* (i.e., *s_i_* regulates *t_j_*). We show that the reachability profile can help us understand how different disorders that alter the cellular functions based on the signaling patterns of the gene regulatory networks. We particularly focus on leukemias, which is challenging due to the heterogeneity of the transcription patterns.

**Summary of related work.** The problem of computing reachability in uncertain network topologies has drawn significant attention in the context of network reliability. Various exact methods, as well as approximate methods, have been proposed. We refer interested readers to several surveys on the topic (Aggarwal *et al.*, 1975; Hwang *et al.*, 1981). Theoretical results on the complexity of the problem reveal that it is #P complete (Brown and Colbourn, 1996; Husfeldt and Taslaman, 2010; Provan and Ball, 1983). The problem is significantly simplified in the case of acyclic graphs. This type of graphs can be represented as Bayesian Networks, for which various inference algorithms exist. However, for this simple case sophisticated inference algorithms are unnecessary. In the context of biological networks, the problem for general graphs was first addressed by Ourfali *et al.* (2007). The goal of these authors was to infer the structure of the signaling network that best explains a set of gene knockout pairs, given a protein–protein interaction network. To achieve this goal, they developed a method to compute the reachability probability for each knockout gene pair. Their method is an exact solution based on the inclusion–exclusion principle (van Lint and Wilson, 1992). However, due to its high time complexity, this method works accurately only for very small networks (i.e., those with a few tens of nodes). PReach (Gabr *et al.*, 2013) computes the exact reachability probability based on polynomial multiplication. It is significantly faster than the inclusion–exclusion method of Ourfali *et al.* (2007) for networks where there are many paths. However, it does not scale to large networks. *Thus, the existing solutions cannot be used to study entire TRNs, and there is a great need for accurate yet efficient methods.*

**Contributions.** Here, we develop a novel method that computes the probability that a signal originated at a given source gene can reach to a given target gene in a given probabilistic network. Unlike existing methods, our solution is both precise (i.e., it computes this probability without error) and it scales to large networks. Our method follows a divide-and-conquer approach. We partition the given probabilistic network into a sequence of loosely connected clusters of nodes. On the boundary between two such consecutive clusters lies a set of nodes called *node separators*. Any signal which originates from the source node and arrives at any node in the latter cluster must visit the node separators. Similar to PReach (Gabr *et al.*, 2013), we model the given probabilistic network using polynomials. The form of the polynomials of our method however differs from that of PReach in a way that allows us to collapse the polynomial to very small size that is determined by the size (number of interactions) of the clusters and the number of nodes in a given boundary. Each term in our polynomial evaluates the existence probability of a collection of subsets of interactions. In brief, instead of computing the reachability probability from the given source node to the target node, we incrementally compute the reachability probability from the source node to each node separator in sequential order. That allows us to avoid storing a massive fraction of terms of the polynomial (i.e., the terms corresponding to the nodes in earlier clusters). Our experimental results on real and synthetic datasets demonstrate that our method scales to very large network sizes while the inclusion–exclusion method (Ourfali *et al.*, 2007) and PReach (Gabr *et al.*, 2013) fail. We also observe that the reachability profiles provide a valuable resource for characterizing leukemias and differentiating the centrality of the genes across different leukemias as well as healthy control groups.

In summary, the key contributions of this work are:

- We introduce a new quantity for evaluating the state of a biological network, the *reachability profile.*
- We introduce a novel, fast and scalable method to compute the reachability profile of large networks, based on polynomials and *polynomial collapsing operators*.
- We demonstrate the usefulness of reachability profiles in *detailed analysis of different types of leukemias*.

The rest of the article is organized as follows. Section 2 describes our method. Section 3 presents our experimental results. Section 4 concludes with a brief discussion.

## 2 METHOD

In this section we present our method in detail. We first define the essential theoretical concepts in Section 2.1. We then present an overview of our method in Section 2.2. We discuss how to compute intermediate reachability probabilities in Section 2.3. We elaborate on how to partition the network in Section 2.4.

### 2.1 Preliminary definitions

We start by formally defining the probabilistic network concept. We provide a list of notations used throughout the article in the Supplementary Material.

**Definition 1 (probabilistic network)**

A probabilistic network *is a graph G=(V,E,P), where V is the set of nodes, E is the set of edges and P:E→(0,1] is a function that associates to each edge a probability value.*

In our context, each node in *V* represents a gene, each edge in *E* represents an interaction between two genes and *P* associates to each edge the probability of the existence of the interaction it represents. For instance, in Figure 1 (top figure), *V* = {*a*,*b*,*c*,*d*} and *E* = {*e*_1_,*e*_2_,*e*_3_,*e*_4_}. We assume that each edge exists independent of all other edges. This assumption is commonly used in the literature for similar problems (Ceol *et al.*, 2010; Szklarczyk *et al.*, 2011). We limit our description to directed networks, although undirected networks can be dealt with by replacing each undirected edge with two edges in opposite directions.

Given a probabilistic network G=(V,E,P), we call the deterministic network *G* = (*V*,*E*) the *maximal deterministic network* of G. In other words, the maximal deterministic network is the deterministic network in which all possible interactions of G are present.

The computational problem we address in this article is: given a probabilistic network, G=(V,E,P), a source node s∈V and a target node t∈V, what is the probability that the *t* can be reached from *s*?

Next, we define a graph concept, *node separator*, which will help us in explaining our method.

**Definition 2 (Node Separator)**

*Let G = (V,E) be a deterministic network and *s,t∈V *be two of its nodes. An s-t node separator of G is a set of nodes *K⊆V *whose removal disconnects t from s in G.*

**Fig. 2.**
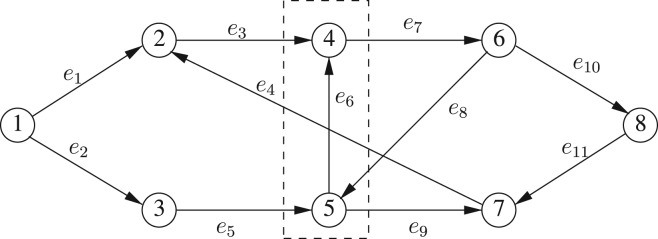
A network with an *s*-*t* node separator. The source is node 1 and the target is node 8. The dotted rectangle indicates an *s*-*t* node separator

Figure 2 illustrates this concept. Here, the source node *s* and the target node *t* are labeled with 1 and 8, respectively. The set of nodes {4, 5} is an *s*-*t* node separator. We say that a node separator is *minimal* if none of its proper subsets is a node separator.

A node separator partitions the nodes of that network into three disjoint subsets:

1. The *left nodes* are the nodes that are reachable from the source, but the target cannot be reached from any of them without going through the node separator (e.g., nodes 1, 2 and 3 in Figure 2, for the node separator {4, 5}).
2. The node separator itself (e.g., nodes 4 and 5 in Fig. 2).
3. The *right nodes* are the remaining nodes (e.g., nodes 6, 7 and 8 in Fig. 2). Notice that these are the nodes from which the target can be reached, but they are not reachable from the source without passing through the node separator.

A node separator *K* also partitions the edges of the given network into three subsets:

1. The *left edges* are the edges between the nodes in the union of left and separator nodes (e.g., edges *e*_1_,*e*_2_,*e*_3_,*e*_5_ and *e*_6_ in Fig. 2). We denote the set of left edges with *L*(*K*).
2. The *right edges* are the edges between right nodes or from a separator node to a right node (e.g., edges *e*_7_,*e*_9_,*e*_10_,*e*_11_ in Fig. 2).
3. The *backward edges* are the edges from right nodes to the separator nodes or from right nodes to left nodes (edges *e*_4_,*e*_8_ in Fig. 2).

**Theorem 1**

*Let G = (V,E) be a deterministic network. Given two nodes, *s,t∈V*, let K be an s-t node separator. For any right node u of K, it is guaranteed that K is also an s-u node separator.*

We prove Theorem 1 in the Supplementary Material.

If a node separator does not yield backward edges, we call it a *good node separator*. We only use good node separators in the rest of the article. So, in what follows by node separator we refer to a good node separator, unless otherwise specified. Finally we define the concept of subset reachability in probabilistic networks.

**Definition 3 (Subset Reachability)**

*Let *G=(V,E,P) *be a probabilistic network. Let s and t (*s,t∈V*) be source and target nodes in *G*. Consider two s-t node separators K_i_ and K_j_ of *G *such that for all nodes *$u\in K_{j}\setminus K_{i},u$ *is a right node of K_i_. Let S and T be two subsets of K_i_ and K_j__,_ respectively. We say that K_j_ is T-reachable from S if all nodes in T are reachable from at least one node in S and none of the nodes in *$K_{j}\setminus T$ *is reachable from any node in S. We denote the probability that K_j_ is T-reachable from S by p(S,T,K_j_).*

### 2.2 Overview of the method

Our method works in two steps.

***Step 1*.** Given a probabilistic network G=(V,E,P) and source and target nodes *s* and *t*, in the first step, we partition G into a sequence of subnetworks that are connected to each other through node separators. In general terms, let us denote the sequence of node separators with *K*_0_, $K_{1},\ldots ,K_{c},K_{c+1}$, where *K*_0_ = {*s*} and *K_c_*_+1_ = {*t*}. We choose these node separators such that ∀i<j, for all nodes $u\in K_{j}\setminus K_{i},u$ is a right node of *K_i_*. Following from Definition 3, the problem we solve in this article is equivalent to computing $p(K_{0},K_{c+1},K_{c+1})=p(\{s\},\{t\},\{t\})$.

***Step 2*.** At this step we compute the reachability probability from *s* to *t*. More specifically, using this notation above, for any *i* (0 < *i* ≤ *c*), we write the probability *p*({*s*},*T*,*K_i_*_+1_) as

(1) $$p(\{s\},T,K_{i+1})=\sum_{S\subseteq K_{i},S\neq \emptyset }p(\{s\},S,K_{i})p(S,T,K_{i+1})$$

The case *i* = 0 is a special one. Since *K*_0_ contains *s*, we have *T* = {*s*}. Thus the probability to reach the source node is 1. Following from Equation (1), our algorithm iteratively computes *p*(*K*_0_,*K_c_*_+1_,*K_c_*_+1_) by moving from one node separator to the next, starting from *K*_0_.

The correctness of Equation (1) follows from the definition of node separator and Theorem 1. More specifically, in order to reach to any node in $T\subseteq K_{i+1}$, we have to visit at least one node in *K_i_*. The product *p*({*s*},*S*,*K_i_*)*p*(*S*,*T*,*K_i_*_+1_) in Equation (1) is the probability that a signal reaches *T* by visiting all the nodes in *S* and no other node in *K_i_*– *S*. The summation in this equation enumerates all possible subsets $S\subseteq K_{i}$. Thus, it is accumulates the probability of all possible alternative routes from *s* to *T* defined by all possible subsets *S*.

**Fig. 3.**
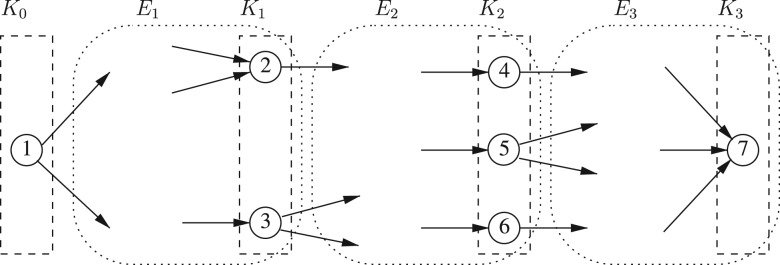
A hypothetical network with two disjoint *s*-*t* node separators, *K*_1_ = {2,3} and *K*_2_ = {4,5,6}. Source and target nodes are labeled with *s* = 1 and *t* = 7. For uniformity, we consider *K*_0_ = {1} and *K*_3_ = {7} also to be node separators

Figure 3 illustrates our method. In this example, the set of edges in *E* is split into three non-overlapping sets using four node separators *K*_0_, *K*_1_, *K*_2_ and *K*_3_ where *K*_0_ = {*s*} and *K*_3_ = {*t*}. These sets are $L(K_{1}),L(K_{2})\setminus L(K_{1})$, and $L(K_{3})\setminus L(K_{2})$. Each of these sets define a subnetwork of G. Once the network is partitioned this way, instead of computing the reachability probability directly from *s* to *t*, we compute it incrementally by advancing from one node separator to the next. For the example in Figure 3, we first consider the separator *K*_1_, then *K*_2_, finally *K*_3_. At each node separator, we only consider the subnetwork which contains the left edges of that separator.

To understand Equation (1) better, consider the separators *K*_1_ and *K*_2_ in Figure 3. There are three possible scenarios to reach to a subset *T* of *K*_2_, say *T* = {4} from *s* = 1. Each of these scenarios corresponds to a nonempty subset of *K*_1_ = {2, 3}.

1. Visit *S* = {2} and do not visit $K_{1}\setminus S=${3}. This happens with probability *p*({1},{2},*K*_1_)*p*({2},{4},*K*_2_).
2. Visit *S* = {3} and do not visit $K_{1}\setminus S=${2}. This happens with probability *p*({1},{3},*K*_1_)*p*({3},{4},*K*_2_).
3. Visit both nodes in *S* = {2, 3}. This happens with probability *p*({1},{2,3},*K*_1_)*p*({2,3},{4},*K*_2_).

The sum of the three probabilities above yields the probability *p*({1},{4},*K*_2_).

In Section 2.3, we explain how we compute Equation (1) efficiently for *i* > 0 (Step 2). In Section 2.4, we explain how we choose the node separators (Step 1).

### 2.3 Computing the reachability probability

In Equation (1), we presented an iterative formula to compute the reachability probability *p*({*s*},{*t*},{*t*}) by splitting the network using cuts *K*_0_, *K*_1_, … , *K_c_*_+1_. Although this equation reduces the scale of the problem to the subnetworks between consecutive cuts, computing it efficiently still remains to be a challenge. Here, we describe how we compute this probability efficiently yet provably correctly. More specifically, given two consecutive node separators *K_i_* and *K_i_*_+1_ (0 < *i* ≤ *c*) and given *p*({*s*},*S*,*K_i_*) for all subsets $S\subseteq K_{i}$, we discuss how we compute *p*({*s*},*T*,*K_i_*_+1_) for all subsets $T\subseteq K_{i+1}$.

From the definition of left edges, we know that the probability *p*({*s*},*S*,*K_i_*) depends only on the edges in *L*(*K_i_*). This is because *L*(*K_i_*) contains all the edges that can lie on a path from *s* to any node in *K_i_*. Let us denote the set of edges in $L(K_{j})\setminus L(K_{j-1})$ with *E_j_* for any 0 < *j* (i.e., left edges of *K_j_* which are also right edges of *K_j_*_– 1_). Thus, the probability *p*({*s*},*T*,*K_i_*_+1_), depends only on the edges in *E_i_*_+1_ when *p*({*s*},*S*,*K_i_*) is given for all *S*. This implies that it is possible to compute the probability *p*({*s*},*T*,*K_i_*_+1_) by considering only the edges in *E_i_*_+1_ when *p*({*s*},*S*,*K_i_*) is known $\forall S\subseteq K_{i}$. Below, we compute this probability by transforming the probabilistic network into a collection of polynomials.

**Transformation into polynomial space.** Assume that the given probabilistic network, G=(V,E,P), contains *n* edges and *m* nodes, denoted with *E* = {*e*_1_,*e*_2_, … *e_n_*} and *V* = {*v*_1_,*v*_2_, … *v_m_*}, respectively. As the first step of the transformation, we associate to each edge a polynomial called the *edge polynomial*. More precisely, for edge $e_{i}\in E$, let *p_i_* = *P*(*e_i_*) and *q_i_* = 1– *p_i_* denote the existence and absence probability of *e_i_*_,_ respectively. We define the edge polynomial of *e_i_* as the first degree polynomial of two variables, *x_i_* and *y_i_*, *F_i_*(*x_i_*,*y_i_*) = *p_i_x_i_* + *q_i_y_i_*.

Consider a subset *E ′* of the edges in *E*. We define the *edge aggregation polynomial* for *E ′*, denoted with *F*(*E ′*), as the product of all the edge polynomials associated with the edges in *E ′*:

(2) $$F(E\;\prime )=\prod_{e_{i}\in E\;\prime }F_{i}(x_{i},y_{i})=\sum_{E\subseteq E\;\prime }\prod_{e_{i}\in E}p_{i}x_{i}\prod_{e_{j}\in E\;\prime \setminus E}q_{j}y_{j}.$$

Notice that each term in the summation above corresponds to one of the possible deterministic configurations for the network topology. The coefficient of the term $\prod_{e_{i}\in E}x_{i}\prod_{e_{j}\in E\prime \setminus E}y_{j}$ in *F* is the probability of observing all the edges in E and not observing any edge in E′∖E. To understand this better, consider the network in Figure 1 (network on the top). In the edge aggregation polynomial of this network, the term *x*_3_*x*_4_*y*_1_*y*_2_ corresponds to the deterministic instance where only edges *e*_3_ and *e*_4_ are present (i.e., bottom left network in Fig. 1). The coefficient of this term is *q*_1_*q*_2_*p*_3_*p*_4_ which is the probability of observing that network instance.

**Reachability in polynomial space.** As we explain in Equation (2), the terms of the edge aggregation polynomial represent different deterministic network configurations. Thus, the probability *p*({*s*},*T*,*K_i_*_+1_) is equal to the sum of the coefficients of a specific subset of the polynomial terms: The terms which yield a topology where *K_i_*_+1_ is *T*-reachable from {*s*}.

At this point, the polynomial transformation seemingly makes the reachability problem as complicated as exhaustively enumerating all network topologies. This is because, (i) the edge aggregation polynomial has as many terms as the number of network topologies; and (ii) finding the subset of polynomial terms which yield the desired topologies will incur additional computational cost. Below, we build a novel algebra on the edge aggregation polynomial to compute this value by enumerating only a tiny fraction of the polynomial terms.

Algorithm 1 presents a pseudocode that describes our algorithm for constructing the polynomial needed to compute *p*({*s*},*T*,*K_i_*_+1_). The algorithm takes the existing edge aggregation polynomial for the edges in *L*(*K_i_*) as input. At each iteration it grows that polynomial by aggregating it with the edge polynomial of a new edge in *E_i_*_+1_ (Step 2). It then reduces the size of the resulting polynomial by collapsing it (Step 3). Briefly, the collapsation step merges all terms which correspond to configurations in which *K_i_*_+1_ is *T*-reachable from *s*, for each possible subset *T* of *K_i_*_+1_, into a single term by replacing the variables in these terms with a single variable denoted with *z_T_*. Thus, the coefficient of *z_T_* is the sum of the coefficients of the original terms that were collapsed. In the rest of this section, we elaborate on these steps, particularly the collapsation step.

**Algorithm 1 Compute the edge aggregation polynomial for *L*(*K_i_*_+1_)**

**Require:** Probabilistic graph G=(V,E,P)

**Require:** Node separators *K_i_* and *K_i_*_+1_

**Require:** Edge aggregation polynomial *F ′*= *F*(*L*(*K_i_*)).

 1: **for all** $e_{j}\in E_{i+1}$ **do**

 2: Aggregate edge polynomial of *e_j_* as *F ′*= *F ′*× *F_j_*(*x_j_*,*y_j_*)

 3: Collapse *F ′*

 4: **end for**

We start by introducing some notation which will simplify our polynomial algebra below. For a subset of edges E⊆E, we denote the set of indices of the edges in E by Ind(E). For instance, for $E=\{e_{2},e_{3},e_{8}\}$, we have Ind(E)={2,3,8}

Let us denote the subset of edges of *E_i_*_+1_ which have been multiplied into the edge aggregation polynomial so far with $E\subseteq E_{i+1}$ and its set of indices with Θ=Ind(E).

Following from Equation (2), since E and *L*(*K_i_*) are disjoint, we can write the edge aggregation polynomial of the edge set $E\cup L(K_{i})$ as $F(E)F(L(K_{i}))$. To simplify our notation of F(E), for all I⊆Θ, we denote $\prod_{i\in I}x_{i}$ and $\prod_{i\in \Theta \setminus I}y_{i}$ with $x_{I}$ and $y_{\Theta \setminus I}$, respectively. We denote the coefficient of $x_{I}y_{\Theta \setminus I}$ with α*_I_*. Thus, we can write the first polynomial as $F(E)=\sum_{I\subseteq \Theta }\alpha_{I}x_{I}y_{\Theta \setminus I}$.

For each node separator *K_i_*, we define a unique collapsing operator and denote it with *ρ_i_*(). This is a linear operator; it acts on the terms of the given edge aggregation polynomial for the edges in *L*(*K_i_*) independently. Briefly, the collapsed polynomial contains a new variable, *z_s_*, for each subset *S* of *K_i_*. The form of this polynomial is $\rho_{i}(F(L(K_{i})))=\sum_{S\subseteq K_{i}}\beta_{S}z_{S}$. In this representation, *z_S_* corresponds to the case where *K_i_* is *S*-reachable from *K*_0_ (i.e., the original source node), and the coefficient *β_S_* is the probability of observing that case. In other words *β_S_* is equal to *p*({*s*},*S*,*K_i_*) in Equation (1). We explain how this operator works and how we compute it in detail later in this section. For the moment, assume that we have already applied it for the edge set *L*(*K_i_*). Therefore we replace the polynomial, *F*(*L*(*K_i_*)) in the product $F(E)F(L(K_{i}))$ with its collapsed version, denoted by *ρ_i_*(*F*(*L*(*K_i_*))).

After multiplying the first polynomial and the collapsed version of the second polynomial, we get

(3) $$F(E)\rho_{i}(F(L(K_{i})))=\sum_{I\subseteq \Theta }\alpha_{I}x_{I}y_{\Theta \setminus I}\sum_{S\subseteq K_{i}}\beta_{S}z_{S}.$$

Since this product includes edge polynomials from the edge set E, we further reduce its size by applying the collapsing operator *ρ_i_*_+1_() on it and thus obtain $\rho_{i+1}(F(E)\rho_{i}(F(L(K_{i}))))$.

Next, we explain how the collapsing operator works. Given two nodes u,v∈V, let π be a path from *u* to *v* in the maximal deterministic network *G* = (*V*,*E*) of the given probabilistic network. Here, by path we mean the set of edges traversed to reach from *u* to *v*. Let *I* be a subset of indices, I⊆{1,…,n}. We define two set indicator functions χ*_u_*_,_*_v_*() and ω*_u_*_,_*_v_*() for the node pair (*u*, *v*). The first one takes the value χ*_u_*_,_*_v_*(*I*) = 1 if there is a path π from *u* to *v* such that Ind(π)⊆I and 0 otherwise. For instance, in Figure 2, χ_1,8_({1,2,5,6,7,10,11}) = 1. This is because {*e*_2_,*e*_5_,*e*_6_,*e*_7_,*e*_10_} forms a path from 1 (source) to 8 (target) and its set of indices {2, 5, 6, 7, 10} is a subset of the input set {1, 2, 5, 6, 7, 10, 11}. Similarly, the second indicator function takes the value ω*_u_*_,_*_v_*(*I*) = 1 if there is a minimal *u*- *v* cut κ such that Ind(κ)⊆I and 0 otherwise. For example, ω_1,8_({2,3,4,5}) = 1, because {*e*_3_,*e*_5_} forms a minimal cut between nodes 1 and 8 and its set of indices {3, 5} is a subset of input set {2, 3, 4, 5}.

Next, we extend the definitions of the set indicator functions χ and ω to multiple source nodes. The extended function χ*_S_*_,_*_v_*(*I*) evaluates to 1 if there is a path π from at least one node *u* in *S* to *v* such that Ind(π)⊆I and 0 otherwise. Similarly, ω*_S_*_,_*_v_*(*I*) evaluates to 1 if for all nodes u∈S there is at least a minimal *u*- *v* cut κ such that Ind(κ)⊆I and 0 otherwise. Formally, we compute these functions as

(4) $$\chi_{S,u}(I)=1-\prod_{s\in S}\left(1-\chi_{s,u}(I)\right)\;\text{and}\;\omega_{S,u}(I)=\prod_{s\in S}\omega_{s,u}(I)$$

Next, we formalize *T*-reachability of the node separator *K_i_*_+1_. For this purpose, we define a new set indicator function *C_S_*_,_*_T_*() which evaluates to 1 only if *K_i_*_+1_ is *T*-reachable from *S*. Otherwise, it evaluates to 0. We compute this function as

(5) $$C_{S,T}(I)=\prod_{u\in T}\chi_{S,u}(I)\prod_{v\in K_{i+1}\setminus T}\omega_{S,v}(\Theta \setminus I).$$

We prove the correctness of Equations (4) and (5) in the Supplementary Materials.

Now we are ready to put all the pieces together and compute the collapsing operator *ρ_i_*_+1_. Recall that each term of the given edge aggregation polynomial indicates a deterministic subnetwork topology for the edges in E, combined with all deterministic topologies of the edges in *L*(*K_i_*) in which *K_i_* is *S*-reachable from *K*_0_, for every $S\subseteq K_{i}$. If that combination ensures that *K_i_*_+1_ is *T*-reachable from *K*_0_, then the collapsing operator *ρ_i_*_+1_ replaces all the variables of that term with *z_T_*. More specifically, consider a term in Equation (3) after the product has been expanded, in the form $\gamma_{I,S}x_{I}y_{\Theta \setminus I}z_{S}$, where $\gamma_{I,S}=\alpha_{I}\beta_{S}$. We compute the collapsing operator *ρ_i_*_+1_() on this term as

(6) $$\begin{array}{l} \rho_{i+1}\left(\gamma_{I,S}x_{I}y_{\Theta \setminus I}z_{S}\right)=\gamma_{I,S}\sum_{T\subseteq K_{i+1}}C_{S,T}(I)z_{T}.\\ \;+\gamma_{I,S}\left(\prod_{T\subseteq K_{i+1}}\left(1-C_{S,T}(I)\right)\right)x_{I}y_{\Theta \setminus I}z_{S} \end{array}$$

The collapsing operator *ρ_i_*_+1_() [see Equation (6)] transforms each term of the polynomial into a single term. The resulting term either contains the variable *z_T_*, where $T\subseteq K_{i+1}$, or remains unchanged. This is because $C_{S,T}$ either takes the value 0 or 1. Thus, *ρ_i_*_+1_() leaves the term unchanged only if $C_{S,T}=0$ for all *T*. When, $C_{S,T}=1$ for some $T\subseteq K_{i+1}$, the coefficient of *z_S_* becomes 0. It returns γ*_I_*_,_*_S_z_T_* in this case. Furthermore, from Equation (5), we know that if $C_{S,T}=1$, then for all *T′≠T* ($T\prime \subseteq K_{i+1}$), $C_{S,T\;\prime }=0$. Thus, the function *ρ_i_*_+1_() returns no other term containing variable *z′_T_*.

Now suppose that a term has collapsed to *z_T_* and a new edge *e_j_* is added in Step 2 of Algorithm 1. From a polynomial point of view, the *z_T_* variable will be multiplied with *x_j_* and *y_j_*, respectively, resulting in two new terms. From the graph reachability point of view, we know that the edges added prior to *e_j_* already ensure *T*-reachability, so *e_j_* does not make any differece: both its presence and its absence lead to reachable graph configurations. In the polynomial, the coeficients of *z_T_x_j_* and *z_T_y_j_* have to be added together to obtain the reachability probability. To take advantage of this observation, we introduce a special multiplication rule for the *z_T_* variables: both *z_T_x_j_* and *z_T_y_j_* are replaced with *z_T_*, for all $e_{j}\in E_{i+1}$, so that their coefficients are added together.

The collapsing operator is very powerful as it ensures that the size of the edge aggregation polynomial never exceeds $2^{\left|K_{i}|+|E_{i+1}\right|}$ in the worst case (i.e., when the indicator function *C_S_*_,_*_T_*() always returns 0 until the last edge in *L*(*K_i_*_+1_) is aggregated). More importantly, it guarantees to reduce the polynomial size down to $2^{\left|K_{i+1}\right|}$ once the edges in *L*(*K_i_*_+1_) are all aggregated. This is a significant improvement as without the collapsation function, the size of the edge aggregation polynomial $2^{\left|L(K_{i+1})\right|}$ after considering *K_i_*_+1_ and it goes up to 2^|^*^E^*^|^ after including all the edges.

**So what is the reachability probability?** After all the edges in *E_i_*_+1_ have been added, all the terms will collapse, and the polynomial will be $\rho_{i+1}(F(L(K_{i+1})))=\sum_{T\subseteq K_{i+1}}\gamma_{T}z_{T}$. When *K_c_*_+1_ = {*t*} is reached, the polynomial will have only two terms: $\gamma_{\left\{t\right\}}z_{\left\{t\right\}}+\gamma_{\emptyset }z_{\emptyset }$. The coefficient γ_{_*_t_*_}_ is equal to the probability that the target node is reachable from the source node. We prove the correctness of our method in the Supplementary Material.

### 2.4 How to choose node separators

Depending on the topology of the maximal deterministic network there can be many alternative sequences of node separators between the source and target nodes. Regardless of how we choose the node separators, our method guarantees to return the correct result. The node separator choice however can affect the size of the intermediate polynomials and thus the running time of our method in two ways. (i) Ideally, each node separator *K_i_* should contain a small number of nodes as it will produce $2^{\left|K_{i}\right|}$ variables of the form *z_S_*. (ii) Each consecutive node separators should contain a small number of edges between them (i.e., *E_i_* should be small). This is because, in the worst case, they yield $2^{\left|E_{i}\right|}$ terms. Finding an optimal sequence of node separators that minimizes the overall computation time is in itself an intriguing area worth investigating. The right balance between the separator size, the size of the edge sets between the separators and the amount of computation we are willing to spend on finding the solution is hard to find. Here, we use a greedy approach to find good node separators.

We consider the first node separator (*K*_0_) to be the source node itself. We determine the next node separator from the current one by considering all nodes that are one edge further from the current node separator. The set of nodes identified in this way is a minimal node separator, but it is not necessarily good, because it may contain nodes with incident backward edges—see Section 2.1. To make it good, we first identify the nodes that have incident backward edges and replace each of them with all the nodes that are reachable from them in one hop. Thus we advance the node separator toward the target keeping it minimal, and stop as soon as we encounter a good minimal node separator. This way, we aim to keep the size of *E_i_* small. We repeat this process to select more good node separators until we reach the target.

## 3 RESULTS

In this section we experimentally evaluate our method. Section 3.1 presents the datasets and the experimental setup. Section 3.2 examines the running time of our algorithm. Section 3.3 presents the reachability profiles obtained with our method. Section 3.4 evaluates gene centrality based on the reachability profiles. Section 3.5 analyses the stability of the human TRN.

### 3.1 Datasets and implementation details

We evaluate our method using both synthetic and real biological networks.

**Synthetic dataset.** We generated the synthetic network dataset using the Barabasi–Albert random network model (Barabasi and Albert, 1999). We chose this model because it is the de facto standard for the scale-free networks, which best describe most biological networks (Jeong *et al.*, 2000; Todor *et al.*, 2012; Yook *et al.*, 2004). We created six sets of random networks. In each set, we created 10 networks with the same number of nodes: 50, 100, 150, 200, 250 and 300, respectively. The number of edges is twice the number of nodes in each network.

**Real dataset.** For experimentation on real biological networks we used the human regulatory network of Gerstein *et al.* (2012). From this network, we selected only the reliable interactions by taking the intersection with those present in the DIP database (Xenarios *et al.*, 2002). The resulting network has 130 nodes and 172 edges. To assess the interaction confidence for each edge in this intersection, we used the logistic regression method used by Sharan *et al.* (2002). This strategy is used often in the literature to compute interaction confidence (Bader *et al.*, 2004; Ourfali *et al.*, 2007; Sharan *et al.*, 2002; Shlomi *et al.*, 2006). We obtained the gene expression data of 575 leukemia patients from Zhang *et al.* (2012). We obtained control gene expression data in early progenitor cells from Laurenti *et al.* (2013). Both control and leukemia expression datasets are normalized using quantile normalization (Amaratunga and Cabrera, 2001). Each leukemia sample in our dataset belongs to one of eight different subtypes of leukemia: hyperdiploid, TCF3-PBX1, ETV6-RUNX1, MLL, Ph, Hypo, T-ALL and Other, or to non-leukemia sample types CD10CD19 and CD34. We do not include samples from the last two categories in our experiments, since they contain only four samples each. We trained eight different logistic regression models, one for each leukemia subtype to compute interaction probabilities for each subtype separately. Also, we classified the early progenitor cell samples into three categories: primitive (hematopoietic stem cells), lymphoid (ETP, MLP, ProB and B_NKpre) and myeloid (the rest of the samples). We trained a different logistic regression model for each type. Thus, we obtained different probability values for the edges of the human regulatory network, depending on the cancer or control group subtype in which the gene expression levels were measured. This in turn results in different reachability probabilities. We identified all the source and all the target genes in our network using the hierarchical decomposition obtained by HIDEN (Gulsoy *et al.*, 2012). This resulted in 9 source genes and 88 target genes.

We used C++, Matlab and R for implementation. We ran our experiments on an AMD Opteron processor with 256 GB of memory and 1.9 GHz speed.

### 3.2 Evaluation of the running time

**Fig. 4.**
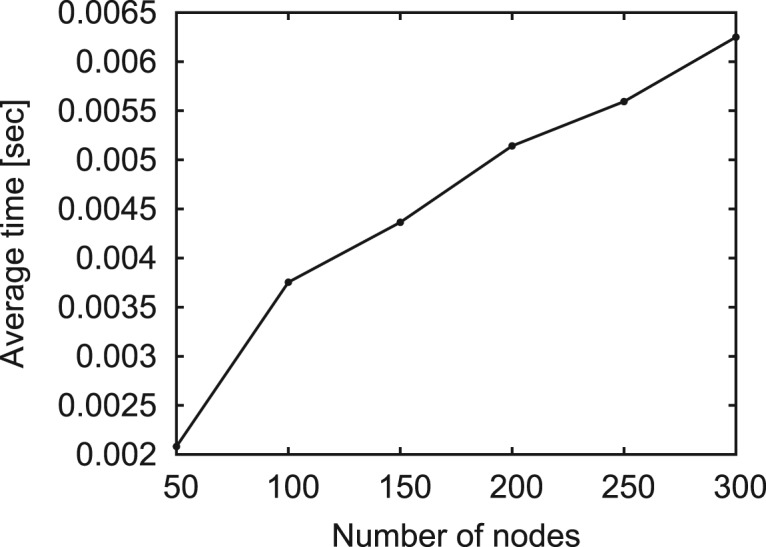
Average running time of our method on Barabasi–Albert networks for growing network sizes

In order to evaluate the performance of our method systematically, we ran it on the synthetic networks of different sizes. We measured the running time for each synthetic network and each source–target pair. We have taken each node, in turn, as a source an then as a target. Thus, computing the reachability profile for the largest network size requires 300 × 299 = 89700 reachability probability computations per network. In total, we computed the reachability profile for 10 × 6 = 60 networks, for a total of 2264500 reachability probabilities. In Figure 4, we report the average running time to compute the reachability probability for one source–target pair for each set of networks. We report the average running time over all networks in the set and over all source–target pairs.

The figure shows that the running time of our method in a scale-free network grows at most linearly in terms of number of nodes. Even for networks as large as 300 nodes and 600 edges, the average running time of our method per source–target pair remains in milliseconds. *This small running time allows us to compute the entire reachability profiles in practical time for a large number of networks, which was not possible before.*

For comparison, the inclusion/exclusion method (Ourfali *et al.*, 2007) and PReach (Gabr *et al.* 2013) fail to complete execution on the same dataset because they exhausted the 256 GB of memory available in the system even for a single source–target node pair of the smallest network in our dataset.

For the real dataset investigated in this article, we computed 11 reachability profiles, one for each leukemia or control group subtype. For each subtype, we computed 9 × 88 reachability probabilities (for 9 sources and 88 target nodes), thus 8712 probabilities in total. Our method computed each of these probabilities in only 2.5 s on the average. Both PReach and the inclusion–exclusion method fail to scale to this network size.

### 3.3 Reachability profiles in the human TRN

**Fig. 5.**
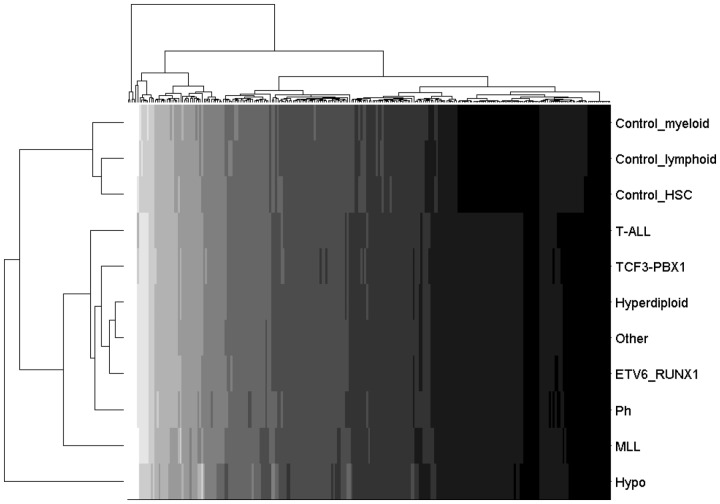
Reachability profiles in the human regulatory network. Each row represents a cancer type or a control group. Each column represents a source–target pair. The intensity of each cell represents the reachability probability for that source–target pair—lighter color means higher probability

For each leukemia or control network, we computed the reachability probability for each pair of source–target nodes. We call this the reachability profile of the network. In Figure 5 we show the reachability profiles for all leukemia subtypes and control groups in a heat map. Each row in the figure represents a leukemia subtype or a control group, and each column represents a source–target pair. The color intensity at a location represents the reachability probability for that pair. We applied hierarchical clustering on both dimensions based on the reachability profiles. Hierarchical clustering correctly clusters the control groups subtypes together, as well as all the leukemias. This shows that the reachability profile can distinguish between healthy and leukemia cases.

Source and target gene groups that show a noticeable gap between their reachability probabilities in control versus leukemia cases include SPI1, POU2F2 as sources and TOPBP1, TFDP1, TFDP2, HDAC1, CDK8, REL, RELA and NFKB2 as targets. While these sources and targets have low a reachability probability for control groups, they exhibit a higher range in leukemia subtypes.

Our findings resonate with earlier observations. Our method clusters the hyperdiploid and the ETV6-RUNX1 subtypes together, while in (Zhang *et al.*, 2012), Supplementary Figure S22, a significant number of genes exhibit similar expression levels in these subtypes. They are frequently studied together, as they are both related to a favorable prognosis in children (Liang *et al.*, 2010; Paulsson *et al.*, 2010). On the contrary, the Hypo subtype, which is least similar to Hyperdiploid and ETV6-RUNX1 in our results, is associated with poor outcome (Holmfeldt *et al.*, 2013).

**Fig. 6.**
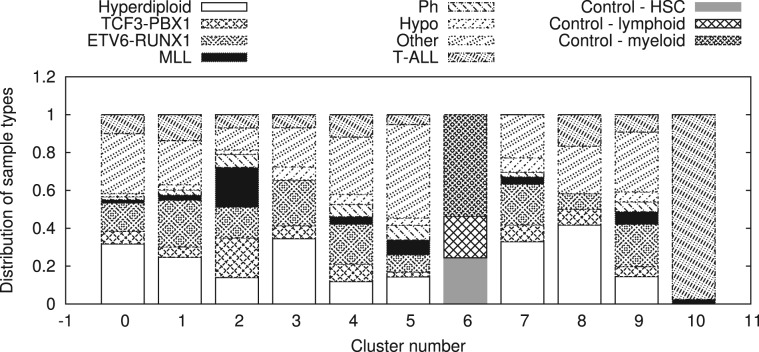
Distribution of leukemia subtype and control group samples within clusters obtained from transcription data alone. Each cluster is normalized by the number of samples it contains

To further appreciate the value added by the reachability profiles to our results, we performed another experiment based solely on gene expression data, without taking the regulatory network into account. In this experiment, we clustered the gene expression samples using *k*-means clustering. We set *k* = 11, as there are totally 11 subtypes in our dataset. Then, within each cluster, we examined the distribution of each leukemia type. The results are shown in Figure 6. Our results demonstrate that, with the exception of cluster 10, consisting primarily of T-ALL samples, all the clusters are a heterogeneous mix and do not have a definitive dominant leukemia type. Although one cluster consists only of control samples, the control subtypes are mixed together. Furthermore, the myeloid subtype samples are spread out through the rest of the clusters. *We conclude that clustering based on gene expression alone is insufficient for classifying leukemia types.*

*In light of these experimental observations, reachability profiles prove to be a reliable and valuable tool for assessing the viability of TRNs working as a whole.*

### 3.4 Gene centrality using reachability profiles

We further illustrate the usefulness of reachability profiles by analysing the centrality of genes based on their contribution toward the reachability profile (Gabr and Kahveci, 2013). For this experiment, we compare the reachability profiles for the original network with the reachability profiles obtained by eliminating one gene from the network. Thus, for each gene, we compute its centrality by comparing the reachability profile for the original network with the reachability profile obtained when the gene is missing. For a given gene *g*, whose centrality is under consideration, and a given source–target pair, the difference in reachability probability can be seen as the probability that the source–target pair is indispensable for connecting the source to the target; in other words, {*g*} is a node separator. Then the sum of this value over all source–target pairs is the average number of source–target pairs for which *g* is indispensable. To formalize this description, let us denote the set of source and target genes with *S* and *T*, respectively. We also denote the probability that gene *t* is reachable from gene *s* in the original network with *p*(*s*,*t*) and the same probability for the network where gene *g* is removed with $p_{\bar{g}}(s,t)$. The centrality of gene *g* is defined as $\sum_{s\in S}\sum_{t\in T}p(s,t)-p_{\bar{g}}(s,t)$.

**Fig. 7.**
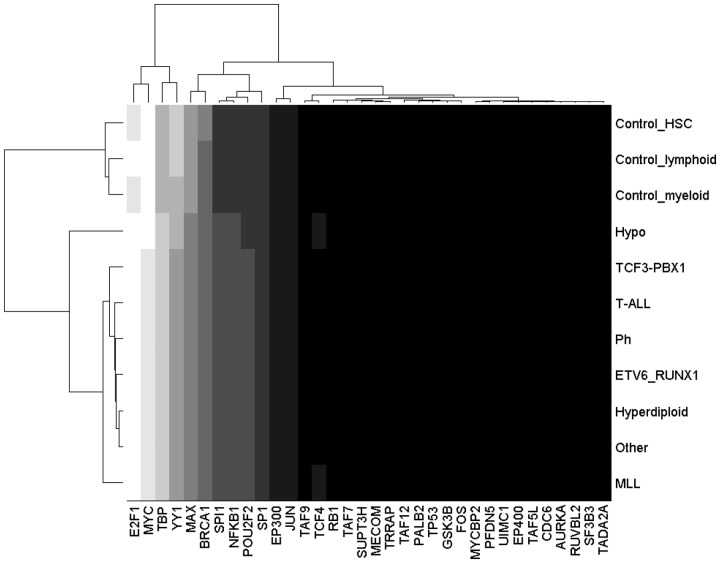
Centrality of genes in the human regulatory network for different leukemia subtypes. Light color denotes high centrality. We only show the genes with centrality value >1 for at least one network

Figure 7 plots the centrality values for each leukemia type and each gene. We excluded from the plot the genes having centrality smaller than 1. As expected, only a few genes have a high centrality, which is a characteristic of scale-free networks. We also performed hierarchical clustering of the leukemia subtypes and of the genes based on their centrality. We observe that the most similar subtypes of leukemias are T-ALL and Ph. The Ph subtype is a chromosomal abnormality resulting from the same translocation found in ALL (Talpaz *et al.*, 2006). The least similar to the first two is Hypo, like in the reachability profiles experiment. TP53 and RB1 are two of the most central genes identified by our method. They are both characterized by alterations in Hypodiploid ALL (Holmfeldt *et al.*, 2013). We see that the most central gene is E2F1, which a transcription factor known to have a crucial role in cell cycle and tumor suppression (Neuman *et al.*, 1996). Thus, malfunctioning of this gene severely affects many pathways in the regulatory network. Likewise, the following two reachable genes, MYC and TBP are known hubs regulating important functions. MYC is involved in cell proliferation and its persistent expression is common to many cancers (Nesbit *et al.*, 1999), while TBP is related to RNA polymerase II, an essential element of DNA transcription initiation (Kornberg, 2007). Among the top genes we identified based on their centrality is also EP300, a histone-modifying gene which was reported to inactivate lesions disrupting hematopoietic development in ETP ALL (Zhang *et al.*, 2012).

### 3.5 Assessment of network stability

Beside characterization of single genes using centrality, we also performed and experiment to characterize the entire human TRN. In this experiment, we assess the level of stability of each of the studied networks. We measure the stability of the network as the average change in reachability probability when edge probabilities are randomly perturbed.

Consider the given probabilistic network G=(V,E,P) and the sets of source and target nodes *S* and *T*. Also consider a parameter δ that denotes the maximum change in edge probabilities. We defined a perturbed edge probability function *P*^δ^ → [0,1] that, for each edge e∈E, returns a value drawn uniformly at random from the range P(e)±δ∩[0,1]. We constructed a perturbed network $G^{\delta }=(V,E,P^{\delta })$. For every pair of s∈S and t∈T, we measured the reachability probability in *G* as *p*(*s*,*t*), as well as that in *G*^δ^ as *p*^δ^(*s*,*t*). We then computed the absolute difference $\left|p^{\delta }(s,t)-p(s,t)\right|$. We repeated this experiment 20 times. We computed the average of the resulting values over all s∈S and t∈T, as well as over the 20 experiments.

**Fig. 8.**
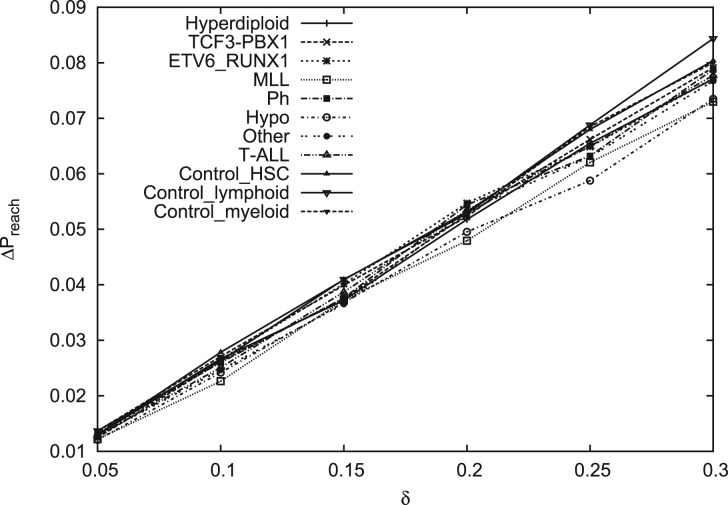
Effect of random perturbation as a measure of network stability: average change in reachability probability (Δ*P*_reach_) when each interaction probability *p* is altered to a random value in the window *p* ± δ∩[0,1]

We plotted the results for different values of δ for the leukemia networks as well as for the control networks. Figure 8 shows the results. The first observation we draw from the figure is that the change in reachability probability for all networks is linear. We also observe that even by perturbing the edge probabilities in the range of ±0.3, the change in reachability probability does not exceed 0.1. From these two observations we can judge transcription-factor regulation in homo sapiens as highly stable and insensitive to random perturbation. This conclusion holds for both healthy people and leukemia patients. However, we also observe that the gap between the networks is not constant; it slightly increases with the increase of perturbation level. At the extreme case of ±0.3, the gap is maximum. There, T-ALL show the most sensitivity to this level of perturbation, while Hypo and MLL show the least sensitivity.

## 4 CONCLUSION

In this article we have characterized different types of leukemias based on the state of the regulatory networks in patients affected by this disease. The state is evaluated through reachability profiles. The reachability profile describes the ability of regulator genes to affect the transcription factors. For this we developed a fast, exact method for computing the probability for a signal to reach from a source node to a destination node in a probabilistic network. The rigorous mathematical apparatus, which involves polynomials and polynomial collapsing operators, allows fast execution time, demonstrated in the performance evaluation experiments. Valuable uses of the reachability profiles illustrated in this article include leukemia subtype classification, gene centrality evaluation and regulatory network stability analysis. All these are valuable tools for evaluating the viability of the TRN under varying conditions as a whole, not just limited to individual gene expressions levels. An interesting parallel can be drawn between our solution and Bayesian Network inference. However, as we mentioned in Section 1, this alternative is limited to acyclic networks. We see a possible application of the Bayesian Network alternative in combination with the reduction of strongly connected components to single nodes, but this solution deserves a careful examination by itself.

*Funding*: NSF CCF-1251599, NSF DBI-1262451, NSF IIS-084-5439.

*Conflict of Interest*: none declared.
